# Research status and frontiers in liver cancer immunotherapy: a bibliometric perspective on highly cited literature

**DOI:** 10.3389/fonc.2025.1587252

**Published:** 2025-04-10

**Authors:** Pan Su, Yeqiong Han, Jindong Yi, Yu Hou, Yao Xiao

**Affiliations:** ^1^ Teaching and Research Section of Clinical Nursing, Xiangya Hospital, Central South University, Changsha, China; ^2^ Department of Pulmonology, Children’s Hospital, National Clinical Research Center For Child Health, Zhejiang University School of Medicine, Hangzhou, China; ^3^ Department of General Surgery, Xiangya Hospital, Central South University, Changsha, China; ^4^ International Joint Research Center of Minimally Invasive Endoscopic Technology Equipment & Standards, Xiangya Hospital, Changsha, China; ^5^ National Clinical Research Center for Geriatric Disorders, Xiangya Hospital, Central South University, Changsha, China

**Keywords:** liver cancer (LC), immunotherapy, bibliometrics, CiteSpace, hotspot, frontier

## Abstract

**Background:**

Liver cancer is one of the major causes of cancer-related death in the world. As a breakthrough therapy, immunotherapy had significantly improved the prognosis of patients. However, the current research status and research hotspots in the field of liver cancer immunotherapy still lack systematic review. Based on the bibliometric analysis of highly cited papers, this study intended to reveal the current research status, research hotspots and future research trends in this field.

**Objective:**

The purpose of this study was to analyze the national/regional contributions, authors and institutions cooperation network, keywords clustering and keywords burst analysis of highly cited papers on liver cancer immunotherapy through bibliometrics, so as to clarify the research frontier and development direction, and provide objective data support for future research direction and clinical practice.

**Methods:**

The highly cited papers on liver cancer immunotherapy from the Web of Science core collection up to February 23, 2025 were retrieved, and 232 studies were included. CiteSpace was used to build a knowledge map, analyze the distribution of years, countries, authors, institutions and cooperation networks, and identify research hotspots and emerging trends through keyword clustering and burst detection.

**Results:**

The number of highly cited papers continued to increase from 2014 and reached a peak in 2022. China and the United States had the highest number of publications and the centrality of cooperation networks. The author with the highest number of papers was Llovet, Josep M, whose research direction mainly focused on immune checkpoint inhibitor combination therapy and molecular typing. The author with the highest cooperation network centrality was Duda, Dan G, whose research team focused on tumor microenvironment regulation. Harvard University and the University of Barcelona played an important central role in the institutional collaboration. Keywords analysis showed that immune checkpoint inhibitors, tumor microenvironment and combination therapy were the core of liver cancer immunotherapy. Burst keywords such as cell lung cancer, pembrolizumab, advanced melanoma, blockade, lymphocytes, etc. had revealed the research frontier of liver cancer immunotherapy research.

**Conclusion:**

The research on liver cancer immunotherapy had made multi-dimensional progress, with China and the United States leading the global cooperation. The main research directions were the combination strategy of immunization, the regulation of tumor microenvironment and the exploration of novel targets. In the future, it is necessary to optimize treatment resistance solutions, integrate interdisciplinary resources, and promote the development of precision and personalized treatment.

## 1 Introduction

Liver cancer is a cancer that originates in the liver and was an aggressive tumor that often occurs in the context of chronic liver disease and cirrhosis (1). Hepatocellular Carcinoma (HCC) accounts for 75% to 85% of primary liver cancers (2), and was the fifth most common cancer in men and the seventh most common cancer in women, and the third leading cause of cancer-related death worldwide (3). Traditional treatment methods, such as surgical resection, liver transplantation, local ablation and chemotherapy, had achieved certain efficacy in early stage liver cancer, but the effect was limited for advanced patients (4).

In recent years, immunotherapy, especially the application of Immune Checkpoint Inhibitors (ICIS), had brought new hope for the treatment of hepatocellular carcinoma (5). The rise of immunotherapy stemmed from a deeper understanding of tumor immune escape mechanisms. The discovery of programmed death receptor-1 (PD-1) and its ligand, PD-L1, revealed the mechanism by which tumor cells evaded immune surveillance by inhibiting T cell function (6). Based on this mechanism, PD-1/PD-L1 inhibitors had shown significant clinical efficacy in a variety of solid tumors (7–9). In addition, the research on new immune checkpoints such as FGL1, CTLA-4, TIM-3, and LAG-3 were also constantly advancing, providing more possibilities for liver cancer immunotherapy (10–14).

With the wide application of immunotherapy in liver cancer, the number of related research papers had increased exponentially. However, how to identify high-impact research results from the massive papers and reveal the research hotspots and development trends in this field had become an important topic in the academic circle and clinical practice. As a tool for quantitative analysis of paper data, Bibliometrics can systematically reveal the dynamic changes, research hotspots and knowledge networks in specific research fields (15). CiteSpace can extract important knowledge points, development trends and research frameworks in the field through time series analysis, co-word analysis and co-citation analysis of a large number of papers, helping researchers to comprehensively understand the research status and future development direction of this field (16).

Highly cited papers refer to research results that was cited significantly more frequently than similar papers within a certain period of time. Highly cited papers usually represent a milestone achievement in a certain field or research with important academic value, and its analysis can reveal the distribution characteristics of key theoretical breakthroughs, technical paths and academic influence. Therefore, in this study, CiteSpace, a bibliometric analysis tool, was intended to be used to analyze highly cited papers in the research field of liver cancer immunotherapy, so as to identify core countries, authors and institutions, reveal potential research gaps, hot spots and frontier issues, and provide theoretical support and methodological guidance for future research.

## 2 Data and methods

### 2.1 Data sources

**Figure 1:**
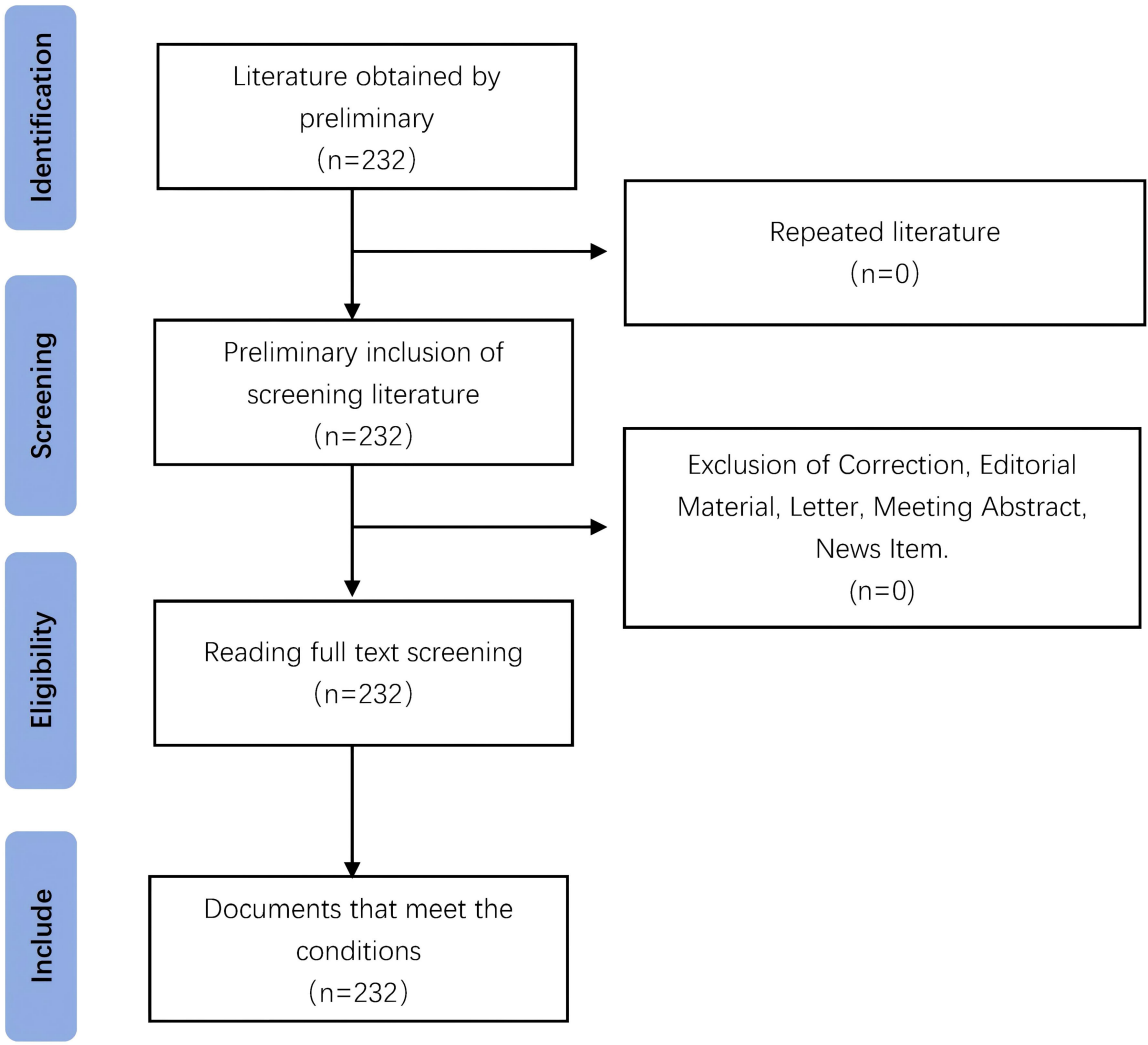
The specific papers screening process.

The advanced search function was used to construct search strategy and search in the WOS database. The time range was set from the establishment of the database to February 23, 2025. The retrieval strategy for WOS core collections was: ((((((((((((((((((((TS=(Hepatic Neoplasms)) OR TS=(Hepatic Neoplasm)) OR TS=(Neoplasm, Hepatic)) OR TS=(Neoplasms, Hepatic)) OR TS=(Neoplasms, Liver)) OR TS=(Liver Neoplasm)) OR TS=(Neoplasm, Liver)) OR TS=(Cancer of Liver)) OR TS=(Liver Cancer)) OR TS=(Cancer, Liver)) OR TS=(Cancers, Liver)) OR TS=(Liver Cancers)) OR TS=(Hepatocellular Cancer)) OR TS=(Cancers, Hepatocellular)) OR TS=(Cancer of the Liver)) OR TS=(Hepatocellular Cancers)) OR TS=(Cancer, Hepatocellular)) OR TS=(Hepatic Cancer)) OR TS=(Cancer, Hepatic)) OR TS=(Cancers, Hepatic)) OR TS=(Hepatic Cancers) AND TS=(immune therapy). Articles and reviews in highly cited papers were selected. The highly cited papers analyzed in this study were directly screened and exported from WOS database, and a total of 232 highly cited papers were retrieved. By reading the title and abstract of the papers, and reading the full text, when necessary. 232 studies were finally included after excluding conference papers, paper call notices, irrelevant topics and duplicate papers. See **Figure 1** for the specific papers screening process.

### 2.2 Methods

Developed by Dr. Chaomei Chen’s team, CiteSpace is a Java-based information visualization tool that can be used to reveal the structure and development trends of scientific knowledge (17). By constructing a scientific knowledge map, the software can analyze research hotspots, frontiers, key papers, core authors and institutions, and predict the development direction of the field (18). This study imported the information of 232 highly cited papers into CiteSpace, set the time span from the establishment of the database to February 23, 2025, and the time partition length was 1 year. The “Top N” strategy was adopted for analysis, Pathfinder was selected by Pruning. The annual distribution of highly cited papers, the distribution and cooperation of countries, authors and institutions, keyword distribution and clustering and emergence analysis will be carried out based on the above strategies. Among them, Top N refers to the nodes whose occurrence frequency is greater than or equal to N every year. Pathfinder is a network pruning algorithm, which is mainly used to remove redundant edges in the network and make the knowledge graph clearer and more concise.Cluster analysis can be used to identify the core subject of a discipline, and Burst keywords can reveal the academic frontier in a specific period. Burst keywords refer to terms or keywords that suddenly and significantly increase in frequency in a specific period of time, usually reflecting emerging trends, hot topics or major breakthroughs in the research field (19–21). In the knowledge map generated by CiteSpace (22), N stands for the number of network nodes, E stands for the number of connections, Density stands for the network density, and Centrality reflects the influence and connectivity of a certain node in the knowledge map, such as country, author, institution, keyword, etc., in the network. The quality of the atlas can be evaluated through Modularity Q (Q value) and Silhouette (S value). Q>0.3 indicates significant cluster structure, S>0.5 indicates reasonable clustering and high homogeneity. The knowledge map uses citation rings to represent the influence of corresponding nodes, and the frequency and line thickness reflect the co-occurrence relationship between nodes.

## 3 Results

### 3.1 Analysis of annual publications

The highly cited papers analyzed in this study were mainly distributed from 2014 to 2024, with an annual publication volume of 1, 6, 10, 8, 16, 19, 24, 25, 52, 47, and 24, respectively, with the highest number of highly cited papers in 2022. No highly cited papers had been detected in 2025, and the reason may be related to our search time.

### 3.2 Countries or regions distribution

China topped the list with 103 papers, followed by the United States with 102 papers. Other countries or regions with high volumes of publications include Spain, Germany and France. The specific results were shown in **Table 1**. In terms of centrality, China leaded with a centrality value of 0.45, demonstrating its centrality in the field. The centrality of the United States was also high, reaching 0.36, followed by the United Kingdom and Germany, the specific results were shown in **Table 1**. The low centrality of countries or regions such as Spain, Italy and Taiwan of China suggested that while their publishing output was significant, their connectivity in global research networks was relatively weak.

**Table 1:** Countries or regions distribution.

Countries or regions	Count	Countries or regions	Centrality
PEOPLES R CHINA	103	ENGLAND	0.36
USA	102	GERMANY	0.22
SPAIN	27	FRANCE	0.18
GERMANY	24	AUSTRALIA	0.14
ITALY	22	CANADA	0.14
ENGLAND	21	USA	0.13
TAIWAN	17	SWITZERLAND	0.13
JAPAN	16	ITALY	0.1
FRANCE	16	JAPAN	0.07
SOUTH KOREA	13	SPAIN	0.06

**Figure 2:**
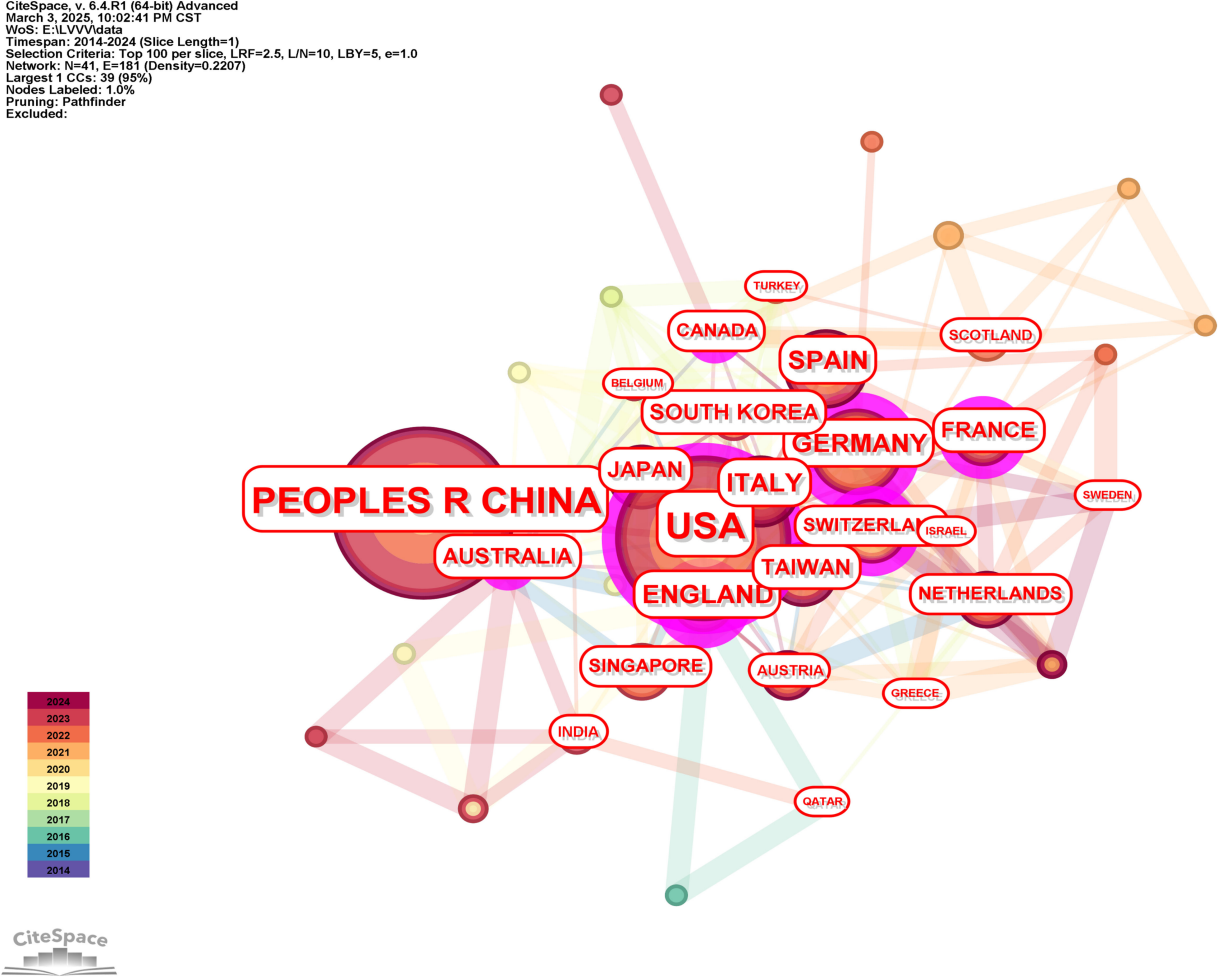
Knowledge map of countries or regions cooperation.

We generated the knowledge map of the national cooperative network through the following strategies (see **Figure 2** for the results): The node selected the country, the Top N selected 100, the Timespan selected 2014-2024, and the Pruning selected the Pathfinder. The results in **Figure 2** show strong collaboration among China, the United States, and European countries or regions such as Germany, Spain, and France, which form an active research core. It was not difficult to see from the knowledge map that countries or regions with higher outputs, particularly China and the United States, also had higher centrality, indicating that they were key players in global collaboration and research dissemination in the field of liver cancer immunotherapy.

### 3.3 Authors distribution

**Figure 3:**
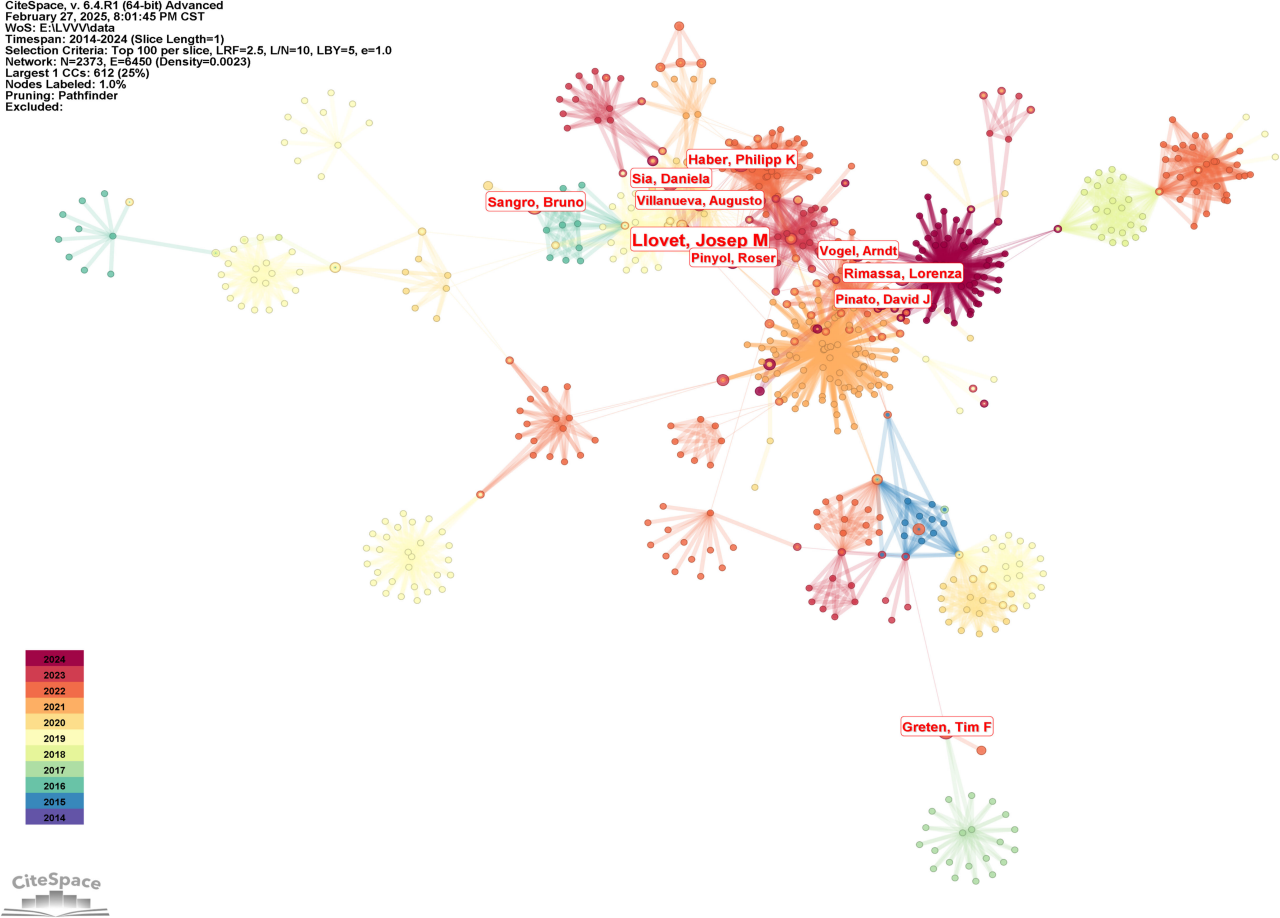
Knowledge map of authors cooperation.

The author cooperation network knowledge map was generated by the following strategies (the results were shown in **Figure 3**): The node selected the author, the Top N selected 100, the Timespan selected 2014-2024, and the Pruning selected the Pathfinder. The result in **Figure 3** shows the collaboration among different authors. The knowledge map reveals multiple groups of authors with a high degree of collaboration, of which Llovet, Josep M and Pinato, David J were central figures in the field, working closely with multiple authors. Other important core authors included Sangro, Bruno, Greten, Tim F, Rimassa, Lorenza, Vogel, Arndt and Haber, Philipp K, among others, with a close network of collaborations among these authors. Among the highly cited authors, Llovet, Josep M, had the highest number of papers with 11, followed by Finn, Richard S, Sangro, Bruno, Sia, Daniela, Haber, Philipp K, Rimassa, Lorenza, Greten, Tim F, Villanueva, Augusto, Pinyol, Roser, Vogel, Arndt. Duda, Dan G had the highest centrality (0.03), followed by Pinyol, Roser, Lujambio, Amaia, Dufour, Jean-Francois, Park, Joong-Won, De giorgi, Ugo, Greten, Tim F, Vogel, Arndt, Galle, Peter R, Melero, Ignacio, the number of authors’ publications and the results of centrality were shown in **Table 2**.

**Table 2:** Authors distribution.

Author	Count	Author	Centrality
Llovet, Josep M	11	Duda, Dan G	0.03
Finn, Richard S	8	Pinyol, Roser	0.02
Sangro, Bruno	6	Lujambio, Amaia	0.02
Sia, Daniela	6	Dufour, Jean-Francois	0.02
Haber, Philipp K	6	Park, Joong-Won	0.02
Rimassa, Lorenza	6	De giorgi, Ugo	0.02
Greten, Tim F	6	Greten, Tim F	0.01
Villanueva, Augusto	5	Vogel, Arndt	0.01
Pinyol, Roser	5	Galle, Peter R	0.01
Vogel, Arndt	5	Melero, Ignacio	0.01

**Figure 4:**
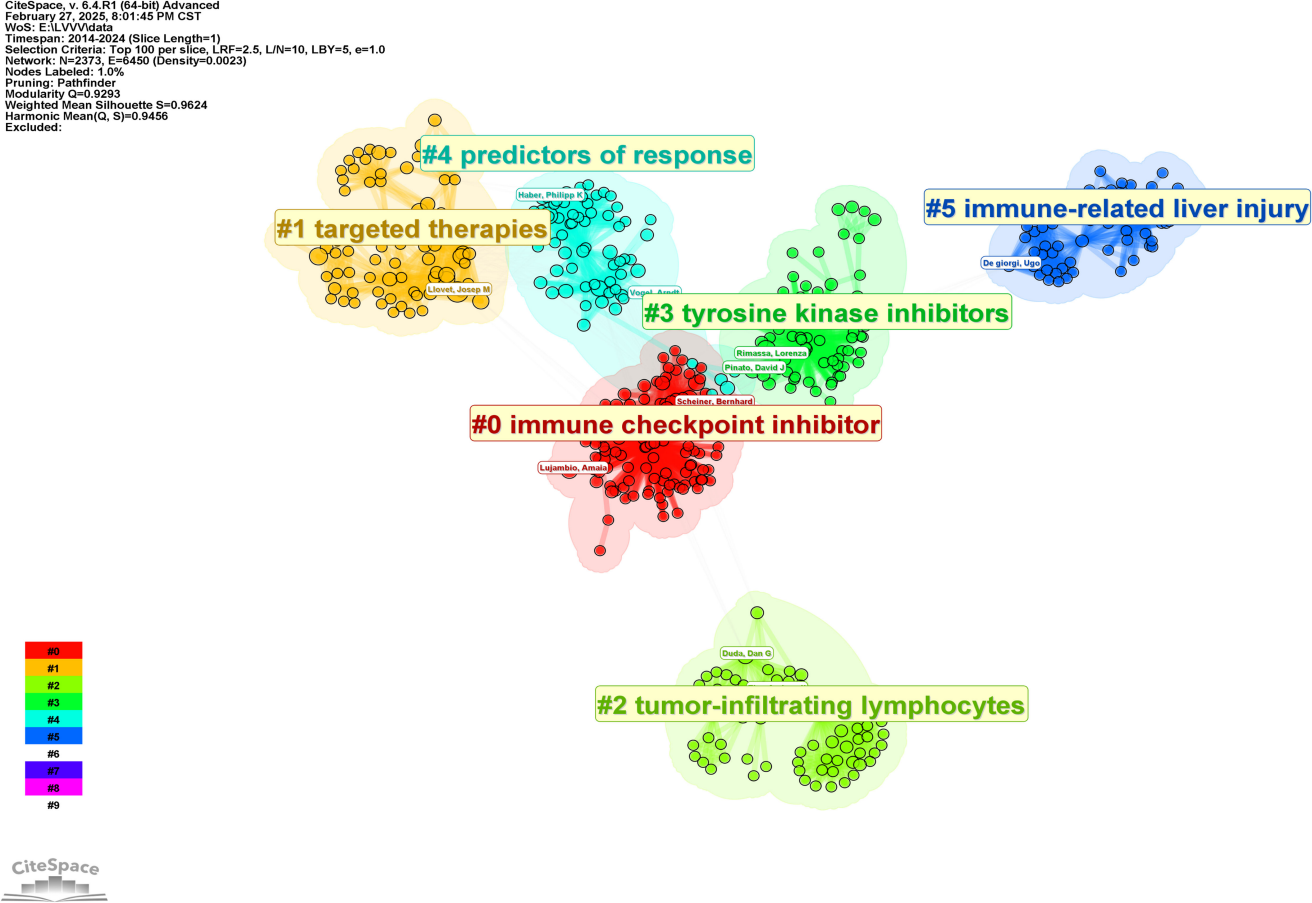
Knowledge map of authors cooperation clustering.

In order to further analyze the research direction of the collaboration between the authors, we conducted cluster analysis for the keywords based on **Figure 3**. The result was shown in **Figure 4**. The Q value is 0.9293 and the S value is 0.9624, indicating a good clustering effect. A total of 6 thematic clusters were obtained. The thematic clustering labels were immune checkpoint inhibitor, targeted therapies, tumor-infiltrating lymphocytes and tyrosine kinase inhibitors, predictors of response and immune-related liver injury, which indicated that the research directions of the authors’ cooperation mainly focused on the above 6 aspects, and the specific clustering information was shown in **Table 3**.

**Table 3:** Authors clustering information.

Cluster ID	Size	Silhouette	Mean(Year)	Top Terms(log-likelihood ratio, p-level)
0	116	0.93	2021	immune checkpoint inhibitor (4.18, 0.05); primary liver cancer (4.18, 0.05); clinical practice guidelines (4.18, 0.05); platinum doublet chemotherapy (4.18, 0.05); hepatitis b (4.18, 0.05)
1	82	0.963	2019	targeted therapies (3.33, 0.1); subtypes (3.33, 0.1); interferon-free regimen (3.33, 0.1); gene expression signature (3.33, 0.1); targets (3.33, 0.1)
2	73	0.99	2019	gm csf (8.75, 0.005); microenvironment (8.75, 0.005); recruitment (4.34, 0.05); angiogenesis (4.34, 0.05); infiltration (4.34, 0.05)
3	73	0.958	2023	antiangiogenic therapy (4.11, 0.05); inhibits tumor growth (4.11, 0.05); systemic treatments (4.11, 0.05); tie2 expressing monocytes (4.11, 0.05); plasmacytoid dendritic cells (4.11, 0.05)
4	69	0.902	2021	predictors of response (3.65, 0.1); stereotactic body radiotherapy (3.65, 0.1); alpha fetoprotein (3.65, 0.1); fatty liver disease (3.65, 0.1); randomized controlled trial (3.65, 0.1)
5	58	0.995	2020	chemotherapy (6.71, 0.01); antibody (6.71, 0.01); durvalumab (6.71, 0.01); nsclc (6.71, 0.01); cs1001 (6.71, 0.01)

### 3.4 Institutions distribution

**Figure 5:**
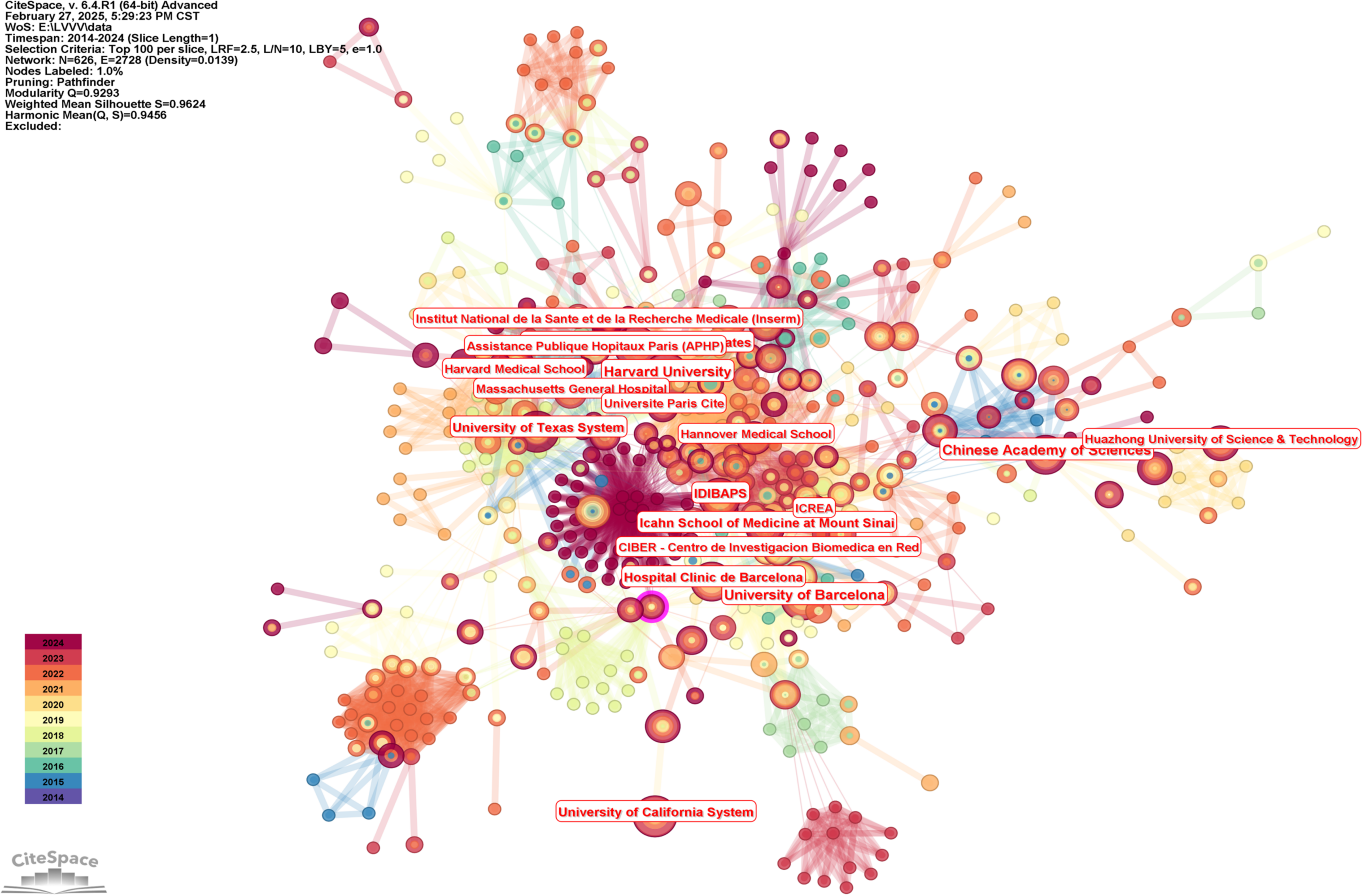
Knowledge map of Institutions cooperation.

We generated the knowledge map of the institutional cooperation network through the following strategies (see **Figure 5** for the results): the node selected institution, the Top N selected 100, the Timespan selected 2014-2024, and the Pruning selected the Pathfinder. In general, the knowledge map center of institutional cooperation network shows a centralized trend, and scattered cooperative groups can be seen around it. Among them, Harvard University, University of Barcelona and Chinese Academy of Sciences occupy the core positions in the cooperation network, demonstrating their importance in the global cooperation network. In terms of publication volume, Harvard University ranked first with 18 papers, followed by the University of Barcelona and the Chinese Academy of Sciences, both with 17 papers. Other institutions with high volume of publications included the Harvard University Medical Affiliate, the University of Texas System, and the University of California System. In terms of centrality, Harvard University’s centrality was 0.2, showing its leading role in the field of liver cancer immunotherapy. Other institutions with high centrality included Fondazione IRCCS Istituto Nazionale Tumori Milan and Hannover Medical School. The University of Sydney and Seoul National University had relatively low centrality. The number of documents issued by institutions and the results of centrality were shown in **Table 4**.

**Table 4:** Institutions distribution.

Institution	Count	Institution	Centrality
Harvard University	18	Fondazione IRCCS Istituto Nazionale Tumori Milan	0.15
University of Barcelona	17	Hannover Medical School	0.1
Chinese Academy of Sciences	17	KU Leuven	0.1
Harvard University Medical Affiliates	15	CIBER - Centro de Investigacion Biomedica en Red	0.08
University of Texas System	15	Columbia University	0.08
University of California System	14	CIBEREHD	0.08
Hospital Clinic de Barcelona	14	University of Sydney	0.07
Icahn School of Medicine at Mount Sinai	14	Chinese Academy of Sciences	0.06
IDIBAPS	14	Seoul National University (SNU)	0.06
Hannover Medical School	12	Cornell University	0.06

**Figure 6:**
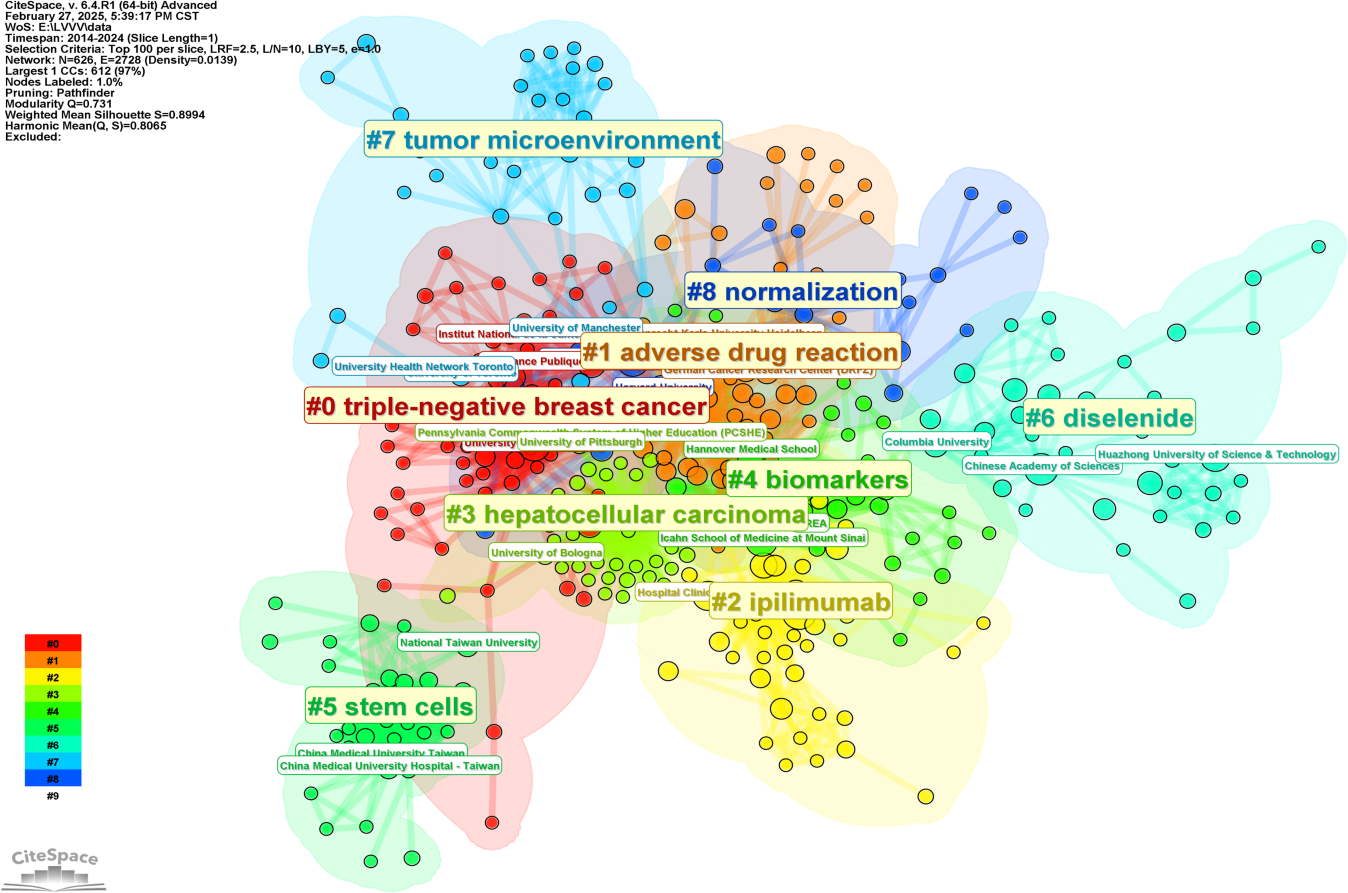
Knowledge map of Institutions cooperation clustering.

In order to further analyze the research direction of the cooperation among the institutions, we conducted cluster analysis for the keywords based on **Figure 5**. The result was shown in **Figure 6**. The Q value of the map is 0.731 and the S value is 0.8994, indicating a good clustering effect. A total of 9 thematic clusters were obtained. The thematic cluster labels were triple-negative breast cancer, adverse drug reaction, ipilimumab, hepatocellular carcinoma, biomarkers and stem cells, diselenide, tumor microenvironment, and normalization showed that the research directions of cooperation among institutions mainly focus on the above 9 aspects. The specific information of clustering was shown in **Table 5**.

**Table 5:** Institutions clustering information.

Cluster ID	Size	Silhouette	Mean(Year)	Top Terms(log-likelihood ratio, p-level)
0	68	0.906	2018	triple-negative breast cancer (6.47, 0.05); paclitaxel (6.47, 0.05); advanced breast cancer (6.47, 0.05); immune checkpoints inhibitors (6.47, 0.05); atezolizumab (6.47, 0.05)
1	68	0.78	2019	adverse drug reaction (4.2, 0.05); toxicity (4.2, 0.05); adverse event (4.2, 0.05); checkpoint inhibitors (4.2, 0.05); side-effect (4.2, 0.05)
2	47	0.876	2018	ipilimumab (6.85, 0.01); hepatoma (3.4, 0.1); tremelimumab (3.4, 0.1); interferon-free regimen (3.4, 0.1); tumour and immune cell area score (3.4, 0.1)
3	45	0.916	2023	hepatocellular carcinoma (hcc) (6.25, 0.05); combination treatment (6.25, 0.05); systemic treatment (6.25, 0.05); tyrosine kinase inhibitor (tki) (6.25, 0.05); immune-related liver injury (3.57, 0.1)
4	44	0.853	2019	biomarkers (4.5, 0.05); targeted therapies (3.45, 0.1); randomized controlled trials (3.45, 0.1); immune-mediated liver disease (3.45, 0.1); precision therapy (3.45, 0.1)
5	37	0.926	2019	stem cells (6.25, 0.05); iron oxide nanoparticles (6.25, 0.05); macrophage (6.25, 0.05); adeno-associated virus (6.25, 0.05); immune-promoting effect (6.25, 0.05)
6	37	0.978	2018	diselenide (4.62, 0.05); biodegradable mesoporous silica nanoparticles (4.62, 0.05); cancer-cell-membrane cloaking (4.62, 0.05); cancer-associated fibroblasts (4.62, 0.05); hepatic arterial infusion chemotherapy (4.62, 0.05)
7	36	0.968	2019	tumor microenvironment (8.62, 0.005); tumor-infiltrating lymphocytes (4.28, 0.05); adoptive t cell therapy (4.28, 0.05); colorectal cancer (4.28, 0.05); drug resistance (4.28, 0.05)
8	26	0.921	2018	normalization (5.55, 0.05); mitochondrial function (5.55, 0.05); hepatic transcriptomics (5.55, 0.05); antiangiogenesis (5.55, 0.05); glucose uptake (5.55, 0.05)

### 3.5 Keywords distribution

**Figure 7:**
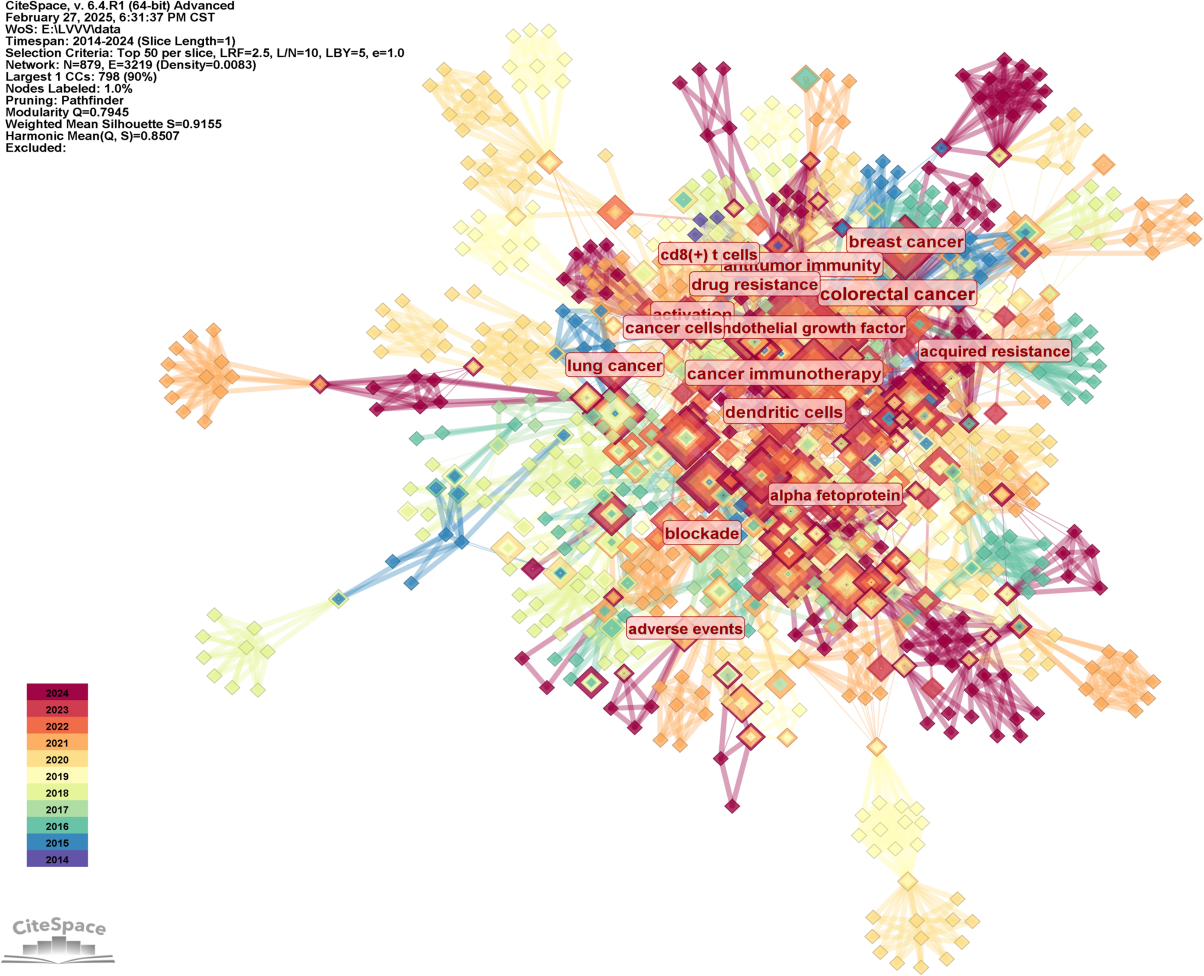
Knowledge map of keywords distribution.

We generated the keyword distribution knowledge map by following strategies (the results were shown in **Figure 7**): the node selected keyword, the Top N selected 50, the Timespan selected 2014-2024, and the Pruning selected the Pathfinder. It was not difficult to see from **Figure 7** that the distribution of keywords was centered, which indicated that the research directions of highly cited authors analyzed in this study were similar. They were working together on some important research topics. The most frequently used keywords were hepatocellular carcinoma (97 times), followed by cancer, double blind, open label, therapy, liver cancer, tumor microenvironment, breast cancer, expression, t cells, etc. The keyword with the highest centrality was colorectal cancer, with a centrality of 0.21, followed by cancer immunotherapy, activation, dendritic cells, antitumor immunity, progression, hepatic stellate cells, cancer cells, promotes. The frequency and centrality of cells, promotes, and keywords were shown in **Table 6**.

**Table 6:** Keywords distribution.

Keyword	Count	Keyword	Centrality
hepatocellular carcinoma	97	colorectal cancer	0.21
cancer	35	cancer immunotherapy	0.16
double blind	26	activation	0.13
open label	25	dendritic cells	0.11
therapy	23	antitumor immunity	0.11
liver cancer	22	progression	0.11
tumor microenvironment	21	hepatic stellate cells	0.1
breast cancer	20	cancer cells	0.1
expression	19	promotes	0.1
t cells	19	cell survival	0.1
cells	19	breast cancer	0.09
sorafenib	18	lung cancer	0.09
suppressor cells	17	in vitro	0.08
regulatory t cells	16	t cell responses	0.08
dendritic cells	15	resistance	0.07
colorectal cancer	15	blockade	0.07
resistance	13	nivolumab	0.07
immunotherapy	13	alpha fetoprotein	0.07
blockade	13	adverse events	0.07
activation	13	intrahepatic cholangiocarcinoma	0.07

**Figure 8:**
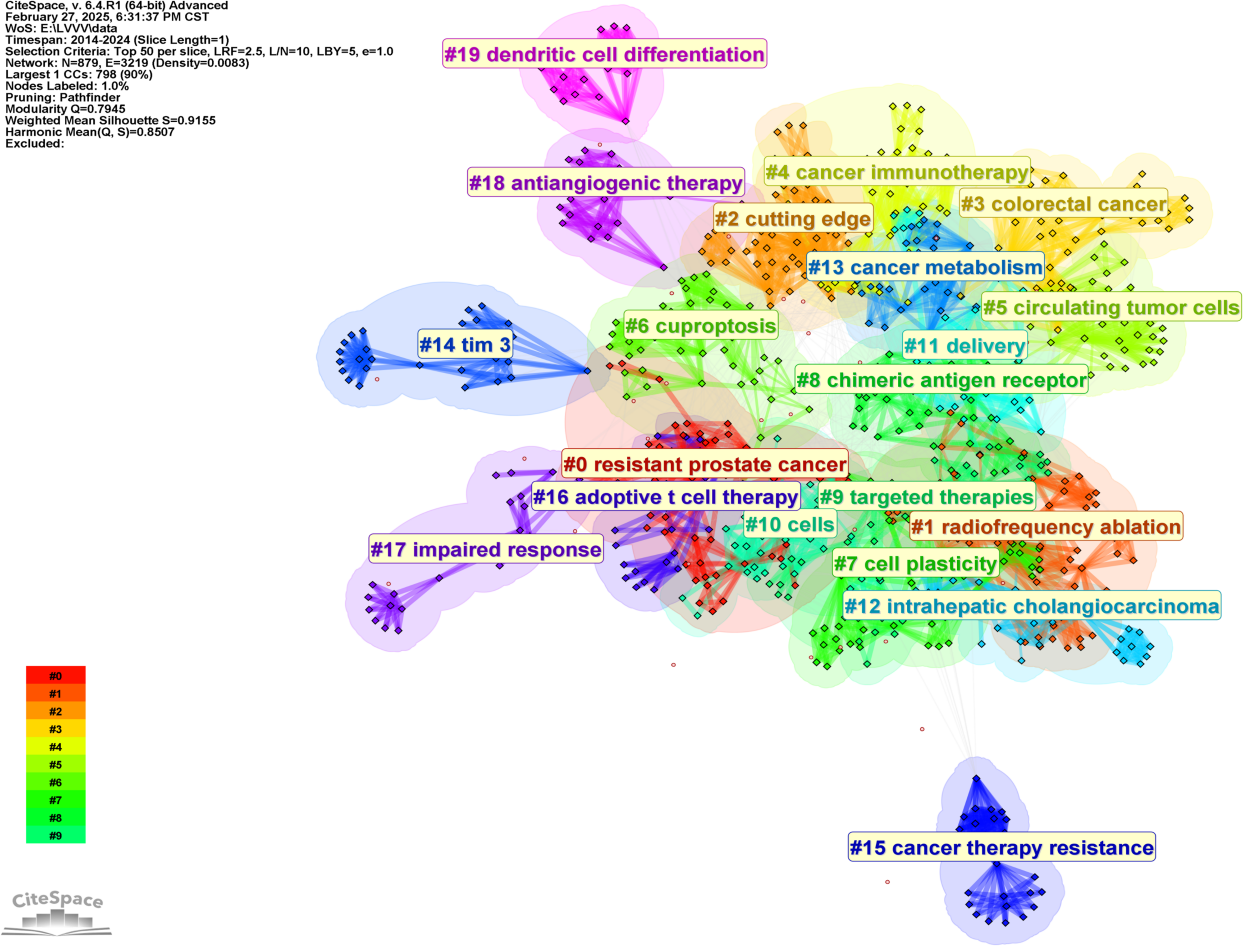
Knowledge map of keywords clustering.

The keywords in **Table 6**, to a certain extent, represent the research direction of the highly cited authors analyzed in this study. In order to further analyze their research direction, we performed cluster analysis on the keywords based on **Figure 7**. The result was shown in **Figure 8**. The Q value of the map is 0.7945 and the S value is 0.9155, indicating good clustering effect. The thematic cluster labels were resistant prostate cancer, radiofrequency ablation, cutting edge, colorectal cancer and cancer immunotherapy, circulating tumor cells, cuproptosis, cell plasticity, chimeric antigen receptor, targeted therapies, cells, delivery, intrahepatic cholangiocarcinoma, cancer metabolism, tim 3, cancer therapy resistance, adoptive t cell therapy, impaired response, antiangiogenic therapy, dendritic cell differentiation, neurodegenerative diseases, and specific results were shown in **Table 7**.

**Table 7:** Keywords clustering information.

Cluster ID	Size	Silhouette	Mean(Year)	Top Terms(log-likelihood ratio, p-level)
0	69	0.92	2017	resistant prostate cancer (12.13, 0.001); immune-related adverse events (8.41, 0.005); pd-1 (6.78, 0.01); adverse drug reaction (6.05, 0.05); bladder cancer (6.05, 0.05)
1	65	0.881	2020	radiofrequency ablation (11.24, 0.001); alpha fetoprotein (11.24, 0.001); stereotactic body radiotherapy (10.37, 0.005); internal radiation therapy (10.37, 0.005); transarterial chemoembolization (10.37, 0.005)
2	55	0.914	2019	cutting edge (7.81, 0.01); tumor growth (7.81, 0.01); cd8(+) t cells (7.81, 0.01); endothelial cells (7.81, 0.01); drug resistance (5.61, 0.05)
3	54	0.906	2019	colorectal cancer (10.84, 0.001); prevent gastric cancer (6.27, 0.05); post-operation (6.27, 0.05); clinical outcome (6.27, 0.05); traditional chinese medicine (tcm) (6.27, 0.05)
4	50	0.88	2018	cancer immunotherapy (6.95, 0.01); adoptive t cell therapies (6.14, 0.05); natural killer cell (6.14, 0.05); brain metastases (6.14, 0.05); sipuleucel t (6.14, 0.05)
5	46	0.902	2019	circulating tumor cells (11.9, 0.001); acquired resistance (11.9, 0.001); gastric cancer (11.9, 0.001); il 6 (5.93, 0.05); infigratinib (5.93, 0.05)
6	45	0.891	2019	cuproptosis (9.86, 0.005); prognostic model (9.86, 0.005); targeted therapy (9.86, 0.005); activation (7.03, 0.01); cell death (7.03, 0.01)
7	44	0.83	2019	cell plasticity (7.28, 0.01); hepatitis b virus (7.28, 0.01); t-lymphocytes (7.28, 0.01); direct-acting antivirals (7.28, 0.01); interferon-free regimen (7.28, 0.01)
8	42	0.891	2019	chimeric antigen receptor (14.92, 0.001); tumor microenvironment (7.55, 0.01); dendritic cells (7.12, 0.01); suppressor cells (6.29, 0.05); epithelial mesenchymal transition (6.29, 0.05)
9	41	0.938	2019	targeted therapies (8.97, 0.005); gene expression signature (6.33, 0.05); aryl hydrocarbon receptor (6.33, 0.05); pathway (6.33, 0.05); transporter atb(0,+) slc6a14 (6.33, 0.05)
10	40	0.894	2019	cells (16.38, 1.0E-4); blockade (14.68, 0.001); resistance (14.59, 0.001); responses (13.54, 0.001); therapy (10.75, 0.005)
11	35	0.896	2018	delivery (10.16, 0.005); non-alcoholic fatty liver disease (9.63, 0.005); nonalcoholic steatohepatitis (8.12, 0.005); liver cancer (6.69, 0.01); checkpoint inhibitor (6, 0.05)
12	30	0.952	2021	intrahepatic cholangiocarcinoma (19.89, 1.0E-4); risk factors (13.22, 0.001); phase ii (9.48, 0.005); extraintestinal manifestations (6.59, 0.05); herpes simplex virus (6.59, 0.05)
13	29	0.899	2019	cancer metabolism (12.13, 0.001); breast cancer (10.21, 0.005); endothelial growth factor (8.41, 0.005); vegf (6.78, 0.01); anti -cancer therapy (6.05, 0.05)
14	28	0.993	2021	tim 3 (9.27, 0.005); rna methylation (9.27, 0.005); circular rnas (9.27, 0.005); cancer therapy (9.27, 0.005); m6a methylation (9.27, 0.005)
15	25	0.998	2019	cancer therapy resistance (11.47, 0.001); mirna-based therapies (11.47, 0.001); mirna-based biomarkers (11.47, 0.001); micrornas (8.7, 0.005); cancer (3.46, 0.1)
16	21	0.971	2017	adoptive t cell therapy (7.84, 0.01); ethnicity (7.84, 0.01); atezolizumab (7.84, 0.01); microrna (7.84, 0.01); cancer-testis antigen (7.84, 0.01)
17	21	0.99	2016	impaired response (9.27, 0.005); antiviral therapy (9.27, 0.005); occupational exposure (9.27, 0.005); immune response (9.27, 0.005); natural history (9.27, 0.005)
18	20	0.97	2019	antiangiogenic therapy (8.75, 0.005); inhibits tumor growth (8.75, 0.005); autophagy (8.75, 0.005); plasmacytoid dendritic cells (8.75, 0.005); macrophage plasticity (8.75, 0.005)
19	19	0.99	2019	dendritic cell differentiation (9.97, 0.005); stat3 phosphorylation contributes (9.97, 0.005); diet induced obesity (9.97, 0.005); idiopathic pulmonary fibrosis (9.97, 0.005); electron transport chain (9.97, 0.005)
20	19	0.985	2022	neurodegenerative diseases (10.44, 0.005); neuroinflammation (10.44, 0.005); transforming growth factor-beta (10.44, 0.005); mesenchymal stem cells (10.44, 0.005); cognitive function (10.44, 0.005)

**Figure 9:**
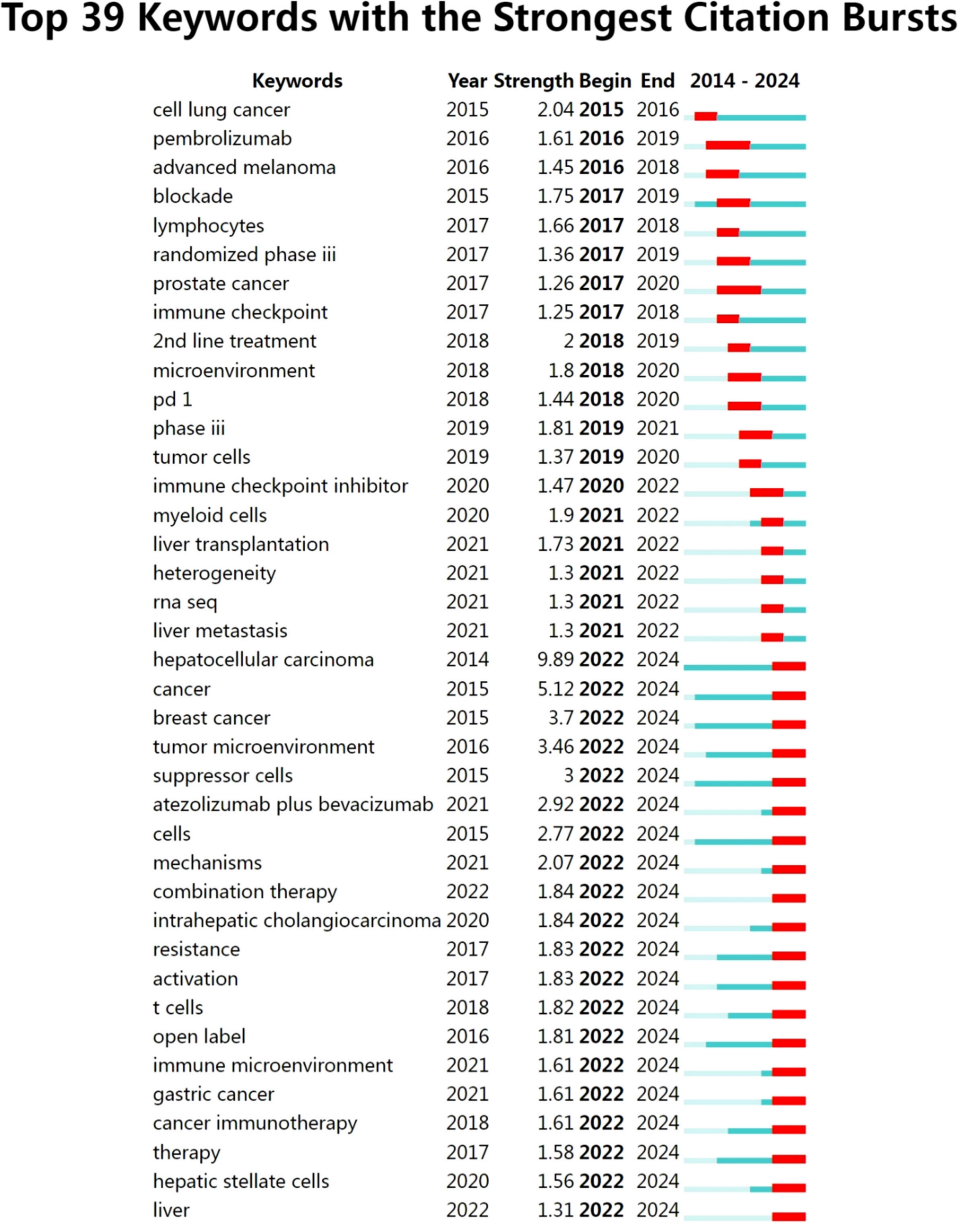
Specific information about burst keywords.

In order to further analyze the research frontiers of highly cited authors analyzed in this study, we conducted keywords burst analysis of keywords, and a total of 39 burst keywords were obtained. They were cell lung cancer, pembrolizumab, advanced melanoma, blockade, lymphocytes, randomized phase iii, prostate cancer, and immune checkpoint, 2nd line treatment, microenvironment, PD-1, phase iii, tumor cells, immune checkpoint inhibitor, myeloid cells, liver transplantation, heterogeneity, rna seq, liver metastasis, hepatocellular carcinoma, cancer, breast cancer, tumor microenvironment, suppressor cells, atezolizumab plus bevacizumab, cells, mechanisms, combination therapy, intrahepatic cholangiocarcinoma, resistance, activation, t cells, open label, immune microenvironment, gastric cancer, cancer immunotherapy, therapy, hepatic stellate cells, liver, and the specific results were shown in **Figure 9**.

## 4 Discussion

### 4.1 Analysis of annual publications

The number of highly cited papers in the field of liver cancer immunotherapy increased year by year since 2014, especially reaching the highest number of published papers in 2022. This trend reflects that liver cancer immunotherapy as an emerging research field had gained increasing attention in recent years. The peak of papers output in 2022 may be related to the publication of several key research results in this field, as well as the clinical application of new immunotherapy approaches such as immune checkpoint inhibitors. With the continuous progress of liver cancer immunotherapy technology, related research results continue to emerge, which promotes the academic community’s attention and investment in this field.

### 4.2 Analysis of Courtiers or regions cooperation

In this study, it was found that China and the United States ranked first in the number of highly cited papers publications in the field of liver cancer immunotherapy. This was mainly due to China’s rich clinical sample resources and rapid development of immunotherapy research system, as well as the long-term advantages of the United States in basic scientific research accumulation, technological innovation and international cooperation network. China, in particular, with its unique clinical samples and advantages in immunotherapy research, had become one of the leading countries in the field globally. The United States, with its long-term research accumulation and strong research and development base, occupied a core position in the global research network. At the same time, countries such as Spain, Germany and France also played an important role in liver cancer immunotherapy research. In addition, from the perspective of centrality analysis, China’s centrality value was 0.45, which was significantly ahead of other countries, indicating its centrality in the global research network. The United States followed closely with a centrality value of 0.36, while the United Kingdom and Germany had a lower centrality, indicating that these countries’ research activities in the field of liver cancer immunotherapy, while important, were less influential in the global collaborative network compared to China and the United States. Through the construction of the knowledge map of the national cooperation network, we further revealed the cooperation model of the world in the field of liver cancer immunotherapy. For example, China had formed close cooperation with the United States and some countries in Europe, which had an important impact on promoting the research and clinical application of liver cancer immunotherapy worldwide.

### 4.3 Research direction analysis of important authors and institutions

The author of the most published paper was Llovet, Josep M. His research mainly focused on the development and application of molecular targeted therapy and immunotherapy for liver cancer. His research team had participated in a number of studies on systematic treatment of liver cancer, especially the clinical trials of targeted drugs and immune checkpoint inhibitors for advanced liver cancer (23). The combination treatment of atizumab (anti-PD-L1) and bevacizumab (anti-VEGF) significantly improved the survival rate of patients in the IMbrave150 trial, becoming a new standard of first-line treatment for liver cancer (9). Llovet, Josep M’s research team was also involved in the molecular classification of liver cancer and precision medicine. Through genomic and transcriptomic analysis, they revealed the molecular heterogeneity of liver cancer and proposed a classification system for liver cancer based on molecular characteristics. For example, one of their studies showed that liver cancer can be divided into different subtypes according to its molecular characteristics, and these subtypes had different responses to different therapeutic strategies (such as targeted therapy and immunotherapy) (24), which provided a theoretical basis for the individualized treatment of liver cancer (25). In addition, the research team of Llovet, Josep M also investigated the combination of local treatments (such as radiofrequency ablation, transarterial chemoembolization TACE) and systemic treatment strategies. One of their studies showed that local treatment can induce the release of tumor antigen and enhance the anti-tumor response of the immune system, thus improving the efficacy of systemic treatment (26). TACE combined with PD-1 inhibitor had shown good efficacy in patients with liver cancer, especially in patients with advanced liver cancer (27).

The author with the highest centrality value was Duda, Dan G. His research direction mainly focused on the interaction between tumor microenvironment and immunotherapy. His research team revealed the heterogeneity of immune cells in TME and its role in immunotherapy through single-cell RNA sequencing and spatial transcriptomics technology (28). Another study of them also showed that the expression levels of immunosuppressive cells (such as myelogenic suppressor cells MDSCs) and immune checkpoint molecules in TME were closely related to the efficacy of immunotherapy (29). The research of the Duda, Dan G team also involved the combination of immunotherapy and targeted therapy strategies for liver cancer. They validated the efficacy of multiple combination treatment strategies through preclinical models and clinical trials. For example, the combination of PD-1 inhibitors with anti-angiogenic agents (such as bevacizumab) had shown significant survival benefits in patients with liver cancer (9). In addition, they investigated the combination of other targeted drugs (such as FGFR inhibitors) with immunotherapy and found that this combination therapy can significantly improve the treatment outcome in patients with liver cancer (30).

Harvard University’s research in the field of liver cancer immunotherapy had formed a global cooperative network, and had established close cooperative relations with many authoritative institutions such as the National Institutes of Health (NIH), the European Association for the Study of Liver Diseases (EASL) and Zhongshan Hospital affiliated to Fudan University in China. Its research scope covered the whole chain from basic mechanism to clinical transformation, and it had made breakthroughs in the following core directions: optimization of immune checkpoint inhibitors (31, 32), interaction regulation of metabolism and immunity (32–35), and development of novel cell therapies (36, 37). Specifically, in terms of immune microenvironment regulation, the Harvard research team had made important discoveries. They revealed that liver cancer cells inhibit T cell function by hijacking glucose metabolism (such as lactic acid accumulation), and that interferon alpha (IFNα) combined with PD-1 inhibitors can reverse this phenomenon, significantly improving the mitochondrial activity and anti-tumor efficacy of CD8+ T cells (38). More interestingly, the team also found that iron death inducers can enhance T cell infiltration by regulating glutathione metabolism, but the risk of hepatotoxicity needs to be carefully balanced (39). In addition, targeting the PPAR-γ pathway reduced the accumulation of myelo-derived suppressor cells (MDSCs), thereby reshaping the immunosuppressive microenvironment (40). In terms of technological innovation, the Harvard team also showed strong research and development strength. For example, nitric oxide nanocarriers (NanoNO) developed at Harvard Medical School were able to consistently release NO and efficiently deliver it to hepatocellular carcinoma. Low-dose NanoNO can not only normalize tumor blood vessels, but also significantly improved the delivery and effectiveness of chemotherapy drugs related to tumor necrosis factor, apoptosis induction, and ligand-based therapy in primary tumors and metastases (41).In addition, the Harvard team also made a breakthrough in the exploration of novel immune checkpoint targets. They revealed that fibrinin-as-protein 1 (FGL1) was the main functional ligand of the immunosuppressive receptor LAG-3. FGL1, which was secreted at low levels in normal liver but was abnormally high expressed in a variety of cancers, inhibits T cell activation and promotes tumor immune escape by binding LAG-3. Blocking FGL1-LAG-3 interaction can significantly enhance anti-tumor T cell responded and produced synergistic effects with anti-PD-1 therapy, providing a potential target for the development of novel cancer immunotherapies (42). At the same time, the Harvard team also delved into the mechanisms of 4-1BB (CD137) as a target for cancer immunotherapy. They summarized the anti-tumor function of urelumab through activation of T cells and NK cells, analyzed the efficacy and hepatotoxicity challenges of urelumab and utomilumab in clinical trials, and proposed strategies such as local administration, bisspecific antibodies and masking techniques to optimize efficacy and reduce systemic toxicity (43). In response to immunotherapy-induced hepatotoxicity, the Harvard team found through single-cell sequencing and animal models that CXCR3+CD8+ effector memory T cells and type 1 conventional dendritic cells (cDC1) were key promoters of response, while myeloid inhibitory subsets were associated with toxicity. Targeting TNFR2 selectively enhanced the anti-tumor immune response while avoiding irAEs exacerbation, which provided a new strategy for the development of safer combination immunotherapies (44). Finally, the Harvard team also systematically summarized the mechanism of action of RING finger ubiquitin ligase (E3s) in cancer and the bidirectional effects of gene variants. By regulating key processes such as cell cycle, DNA repair, signal transduction, and hypoxia response, these enzymes can both promote tumor development as oncogenes (e.g. MDM2) and inhibit cancer as tumor suppressor genes (e.g. BRCA1, VHL). Its dysfunction was closely related to the occurrence of cancer, and had become the focus of targeted therapy (such as small molecule inhibitors), but the clinical efficacy still needs to be further verified (45).

### 4.4 Important research hotspots and frontiers

Based on the cooperative knowledge map and specific clustering information of authors and institutions, and through reading specific papers, we found that the current cooperative research directions in the field of liver cancer immunotherapy mainly focus on the following directions: First of all, the combined treatment strategy of immune checkpoint inhibitors (ICIs) was the core direction. For example, atizumab combined with bevacizumab (IMbrave150 test) significantly extended the overall survival of patients and became the new standard of first-line treatment for liver cancer (9). The combination of PD-1 inhibitors and CTLA-4 inhibitors (such as nabuliumab combined with ipilimumab) had shown a synergistic effect in advanced liver cancer (23). Secondly, the regulatory mechanism of tumor microenvironment (TME) had attracted much attention. Studies had revealed the immunosuppressive effect of myeloid suppressor cells (MDSCs) and immune checkpoint molecules (such as PD-L1) in TME. And reshaped the immunosuppressive environment by targeting metabolic reprogramming (such as the PPAR-γ pathway) or inhibiting angiogenic factors (such as VEGF) (29, 46). Thirdly, the exploration of novel immunotherapy targets had promoted the development of personalized therapy, such as the preclinical studies of LAG-3/FGL1 axis and 4-1BB agonists showing that they can enhance the antitumor activity of T cells (42, 43). Fourthly, the combination of local therapy and systemic immunotherapy had become a hot spot. Radiofrf ablation or TACE combined with PD-1 inhibitors can enhance the systemic immune response by releasing tumor antigens (7), while radiotherapy combined with immunotherapy can improve the therapeutic effect by inducing immunogenic cell death (47). In addition, the development of biomarkers and precision therapy were the key directions of cooperation. Researchers had identified CXCR3+CD8+T cells and CD11c+ antigen-presenting cells in TME through single-cell sequencing and genomic analysis, and explored the application of circulating tumor DNA (ctDNA) in efficacy monitoring (48, 49). Future studies need to further optimize joint strategies, address immune resistance and establish interdisciplinary cooperation networks to promote liver cancer immunotherapy towards precision and individualization.

In the highly cited papers analyzed in this study, Cancer, Immunotherapy, Tumor microenvironment appeared frequently, which may further emphasize the immune escape mechanism and the influence of microenvironment on the immunotherapy effect of liver cancer (50–53). In addition, Double blind and Open label were also high-frequency words, which indicated that randomized controlled trials (RCTs) and open label studies were common research methods for liver cancer immunotherapy at present. However, high-frequency keywords such as Therapy and Sorafenib may indicated that targeted therapeutic drugs played an important role in the research of liver cancer immunotherapy (54). High-frequency keywords T cells, Dendritic cells, Regulatory T cells, etc. may indicated the potential role of these cells in immunotherapy (55–57), especially in immune escape and immune tolerance of liver cancer. The Resistance, Blockade may reflect the challenge of immunotherapy. Keywords Breast cancer and Colorectal cancer also appeared in multiple papers, indicating that liver cancer immunotherapy was closely linked to other cancers (58, 59).

The keywords with high centrality not only reflected the core direction of liver cancer immunotherapy research, but also revealed the hot topics in the current field. Among the high-citation centers analyzed in this study. Colorectal cancer had the highest centrality value, which may be related to the metastatic relationship among colorectal cancer and liver cancer and similar immune escape mechanisms (60, 61). In addition, the centrality of Breast cancer, Lung cancer, and Intrahepatic cholangiocarcinoma was also high, indicating that studies on liver cancer often overlap with immunotherapy studies on these cancer types (28, 62). Tumor microenvironment, Hepatic stellate cells, Regulatory T cells, and Suppressor cells may be involved in the study of tumor microenvironment and immune escape. Cancer immunotherapy, Activation, Blockade, Immunotherapy, Antitumor immunity, T cell responses, Nivolumab mainly reflected the key mechanisms and therapies of immunotherapy (32, 63–65), especially immune checkpoint inhibition, T cell activation, immune escape and drug resistance. Alpha fetoprotein, Resistance, promotion and Cell survival may be closely related to biomarkers and drug resistance in liver cancer immunotherapy. *In vitro* and Cells may involve the design of experiments and the application of research methods in immunotherapy.

Based on the results of keyword distribution and cluster analysis, and subsequent reading of specific papers, we found that the main research directions of liver cancer immunotherapy include the efficacy and mechanism of immune checkpoint inhibitors (ICI) in liver cancer (23, 66, 67), synergistic effect of local ablation and immunotherapy (47, 68), non-alcoholic steatohepatitis (NASH) and immunotherapy resistance to liver cancer (34, 55, 69), molecular typing and targeted therapy of intrahepatic cholangiocarcinoma (iCCA) (24, 70), Ferroptosis and treatment of liver cancer (56, 71), gut microbiome and liver cancer immunotherapy response (72), management of immune-related adverse events (irAEs) (73–75), clinical application of novel biomarkers (such as CRAFITY score) (67, 76), exploration of CAR-T cell therapy in liver cancer (73, 77, 78), regulation of myeloid suppressor cells (MDSC) in the tumor microenvironment (29, 40), tumor metabolic reprogramming and immunotherapy response (involving IDH mutations and glycometabolic pathways) (38, 49, 55), synergistic effects of anti-angiogenic therapy and immunotherapy (e.g., Renvastinib combined with pabolizumab) (7, 23, 79), Application of immunotherapy in liver transplantation patients (80, 81), clinical exploration of neoadjuvant immunotherapy for liver cancer (25, 82), mechanism of T cell depletion and immune checkpoint resistance (44, 48), preclinical study of oncolytic virus combined immunotherapy (41, 83), the immunomodulatory role of tumor-associated fibroblasts (CAF) (84, 85).

Burst keywords that persisted until 2024 included hepatocellular carcinoma, cancer, breast cancer, tumor microenvironment, suppressor cells, atezolizumab plus bevacizumab, cells, mechanisms, combination therapy, intrahepatic cholangiocarcinoma, resistance, activation, t cells, open label, immune microenvironment, gastric cancer, cancer immunotherapy, therapy, hepatic stellate cells, liver. These keywords represented that the research of liver cancer immunotherapy is also developing in the direction of personalized treatment, microenvironment regulation and multi-mode combination therapy, which was also the frontier hot spot of liver cancer immunotherapy. Subsequent studies can further promote the research and development of liver cancer immunotherapy based on these key contents.

Taking the above content into account, it was not difficult to see that the research on liver cancer immunotherapy showed a multidimensional progress trend, covering many aspects such as immunotherapy strategy, tumor microenvironment, cancer types, drug resistance and experimental methods. Immunotherapy research not only focused on the unique immune escape mechanism of liver cancer, but also drawed on the experience of other cancer types to explore how to overcome drug resistance, improve treatment effectiveness, and further delved into the regulation of tumor microenvironment. These research directions provided theoretical support for the development of liver cancer immunotherapy and pointed out the focus of future research.

## 5 Conclusion

In this paper, the bibliometrics method and CiteSpace software were systematically used to comprehensively analyze the highly cited papers in the field of liver cancer immunotherapy, and revealed the research hotspots, international cooperation pattern and future trend in this field. By constructing a collaborative network map of countries, authors and institutions, the central position of China and the United States in global research was clarified, and key authors and their research directions were identified. In addition, through keyword clustering and burst analysis, this paper accurately captured cutting-edge research directions such as immune checkpoint inhibitor combination therapy and tumor microenvironment regulation, providing a new perspective and theoretical support for future research. This multi-dimensional analysis method not only had filled the gap of systematic research in the field of liver cancer immunotherapy, but also provided an important reference for the academic community and clinical practice.

## 6 Limitations

Only highly cited papers related to liver cancer immunotherapy in the Web of Science database were included in this study, and data from other databases were not included at present. The reason for this was that Citespace software can currently only import data from a single database. However, compared with other databases, this software was more efficient in analyzing data in Web of Science (86, 87), which was also the reason why we choose data in Web of Science database for analysis. But to address this limitation, future research could explore integrating data from multiple databases to obtain a more comprehensive and diverse view of research. At the same time, researchers can also consider using other analytical tools or methods to process data from different databases to ensure the comprehensiveness and reliability of research results.

## Data Availability

The original contributions presented in the study are included in the article/supplementary material. Further inquiries can be directed to the corresponding author.
